# Stochastic Time Response and Ultimate Noise Performance of Adsorption-Based Microfluidic Biosensors

**DOI:** 10.3390/bios11060194

**Published:** 2021-06-12

**Authors:** Ivana Jokić, Zoran Djurić, Katarina Radulović, Miloš Frantlović, Gradimir V. Milovanović, Predrag M. Krstajić

**Affiliations:** 1 Institute of Chemistry, Technology and Metallurgy, National Institute of the Republic of Serbia, University of Belgrade, Njegoševa 12, 11000 Belgrade, Serbia; kacar@nanosys.ihtm.bg.ac.rs (K.R.); frant@nanosys.ihtm.bg.ac.rs (M.F.); pkrstajic@nanosys.ihtm.bg.ac.rs (P.M.K.); 2 Institute of Technical Sciences of SASA, Knez Mihailova 35, 11000 Belgrade, Serbia; zdjuric@itn.sanu.ac.rs; 3 Serbian Academy of Sciences and Arts, Knez Mihailova 35, 11000 Belgrade, Serbia; gvm@mi.sanu.ac.rs; 4 Mathematical Institute of SASA, Knez Mihailova 36, 11000 Belgrade, Serbia

**Keywords:** microfluidic adsorption-based sensor, stochastic model, adsorption, mass transfer, ultimate noise performance, detection limit, quantification limit

## Abstract

In order to improve the interpretation of measurement results and to achieve the optimal performance of microfluidic biosensors, advanced mathematical models of their time response and noise are needed. The random nature of adsorption–desorption and mass transfer (MT) processes that generate the sensor response makes the sensor output signal inherently stochastic and necessitates the use of a stochastic approach in sensor response analysis. We present a stochastic model of the sensor time response, which takes into account the coupling of adsorption–desorption and MT processes. It is used for the analysis of response kinetics and ultimate noise performance of protein biosensors. We show that slow MT not only decelerates the response kinetics, but also increases the noise and decreases the sensor’s maximal achievable signal-to-noise ratio, thus degrading the ultimate sensor performance, including the minimal detectable/quantifiable analyte concentration. The results illustrate the significance of the presented model for the correct interpretation of measurement data, for the estimation of sensors’ noise performance metrics important for reliable analyte detection/quantification, as well as for sensor optimization in terms of the lower detection/quantification limit. They are also incentives for the further investigation of the MT influence in nanoscale sensors, as a possible cause of false-negative results in analyte detection experiments.

## 1. Introduction

**Figure 1:**
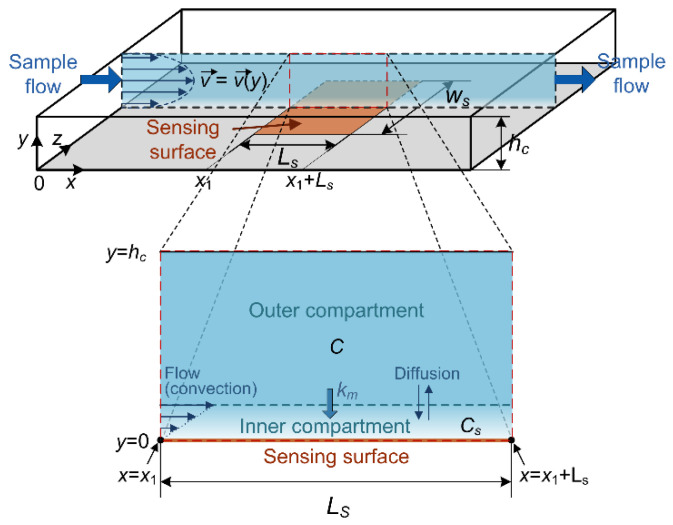
Adsorption-based microfluidic sensor: schematic representation of the typical system geometry with designations of dimensions and coordinate axes. The magnified partial cross-section of the microfluidic reaction chamber in the sensing surface zone is given as an illustration of the two-compartment model approximation of the spatially and time-dependent target substance concentration, affected by coupled adsorption–desorption and mass transfer processes of target analyte particles.

Microfluidic sensors are promising tools for chemical and biological detection [1,2,3,4]. The operation of a large class of such devices, known as adsorption-based sensors, relies on the adsorption–desorption (AD) process of a target substance on the surface of a sensing element. These include SPR (Surface Plasmon Resonance), CNT (Carbon NanoTube) or NWFET (NanoWire Field Effect Transistor), resistive graphene-based, potentiometric, SAW (Surface Acoustic Wave), FBAR (thin Film Bulk Acoustic wave Resonator), microcantilever sensors, etc. [5,6,7,8,9,10,11,12,13]. The sensing element of microfluidic sensors is typically located in a flow-through reaction chamber, where the sample to be analyzed is introduced (Figure 1). The AD process is coupled with mass transfer (MT) processes of target particles in the microfluidic chamber. Via MT processes (convection and diffusion), particles are transported to specific sites on a sensing surface where adsorption occurs, and away from the adsorption sites after desorption. A coupled effect of AD and MT processes determines the temporal change in the number of particles adsorbed on the sensing surface, *N*(*t*), which causes a change in a measurable sensor parameter, yielding the sensor response. Hence, the sensor time response can be considered as determined by the time evolution of the number of adsorbed target particles, *N*(*t*). As it contains information on the target substance presence and its concentration in the analyzed sample, the time response analysis enables both detection and quantification of chemical substances or biological specimens, thus having important applications in environmental protection, public and personal healthcare and security, the food industry, agriculture, and defense. Analysis of response kinetics can provide information as early as in the transient regime, i.e., before the binding process of target particles reaches the steady state, which significantly shortens the time needed to obtain the data. The response kinetics also contains information on the parameters that characterize the interaction process of target particles and surface binding sites [14], thus enabling the characterization of bimolecular binding reactions, important for the fundamental understanding of vital biochemical processes and pharmacology.

The random nature of the AD process coupled with MT causes fluctuations in the number of adsorbed particles, which result in sensor signal stochastic fluctuations known as AD noise, binding/unbinding noise, biological or chemical noise [15,16,17,18,19]. The total fluctuations in the sensor signal also depend on other kinds of noise originating from the sensor transduction mechanism and the read-out circuitry, but the unavoidable AD noise determines the sensor’s ultimate noise performance and poses fundamental detection and quantification limits inherent to all adsorption-based sensors. The contribution of AD noise to the total sensor noise can even be dominant [16,17,20,21]. Thus, the analysis of AD noise and related parameters of stochastic sensor response becomes an important tool for the optimization of adsorption-based chemical and biological microfluidic sensors in terms of reliable analyte detection and quantification, and also in terms of improved sensing performance (i.e., higher signal-to-noise ratio and lower minimal detectable and quantifiable concentrations). This is especially true because miniaturization is a general trend in the field of chemical and biological sensors, focusing on adsorption-based micro- and nanodevices, where achieving a sufficiently high signal-to-noise ratio (SNR) can be a challenge [20]. As AD noise also contains information about the quantity of the target substance in the sample, about binding process parameters, and about substance parameters useful for its recognition [15,22,23,24,25], mathematical modeling and more profound knowledge of AD fluctuations characteristics, both in the transient regime and in the steady state of sensor response, can enable the development of new measurement methods based on stochastic (i.e., noise) analysis in micro/nanosensors, as an addition to the existing conventional methods.

Stochastic mathematical models of sensor response consider the time-dependent number of adsorbed particles as a random process, *N*(*t*), whose expected value reveals the binding (i.e., the sensor response) kinetics, and the variance is a measure of the sensor’s AD noise. As stochastic models take into account the influence of individual events of particle binding and unbinding to the surface adsorption sites on the sensor response, as well as the inherent random nature of these events, they are more accurate in describing the binding kinetics than deterministic models. They describe response fluctuations, which are always present. For the analysis of stochastic sensor response, stochastic simulations are often used. However, analytical approximations of stochastic models are very useful, because they offer a good insight into the dependences of the response statistics on various sensor system parameters, while being more efficient than simulations in which high accuracy requires large computing resources and a long computation time. Stochastic models for the analysis of sensor response should take into account MT effects, as the randomness of the number of adsorbed particles originates from the coupling of the inherently stochastic AD process and MT.

Approximate mathematical models that enable the analysis of statistical parameters (expected value, standard deviation, and variance) of the stochastic time response of adsorption-based (both chemical and biological) sensors and their noise performance metrics (AD noise power spectral density and signal-to-noise ratio) have been developed for some practically significant cases [15,16,18,20,21,24,25,26,27,28,29,30]. Based on these references, it can be concluded that the closed-form solutions for the mentioned quantities are only devised for simplified cases, during the transient regime of the binding process on the sensing surface, or after the steady state of the binding process is reached. For example, in the analysis given in [26], analytical solutions were used for the time-varying expected value and relative fluctuations in the response of an adsorption-based plasmonic sensor, which fluctuate only due to the stochastic nature of the AD process. Analyte transport processes to and from adsorption sites, and the depletion of analyte particles from the sample during adsorption were not taken into account in the closed-form expressions. That corresponds to the idealized situation when MT is fast enough compared to the AD process, and when the number of analyte particles available for adsorption in the sensor’s reaction chamber is much greater than the number of adsorbed particles at any given time, so the particle concentration in the chamber is considered as constant in time and uniform in space. The authors of [20] considered the scaling effects of biosensor systems through the stochastic analysis that takes into account the probabilistic capturing (i.e., adsorption) process. The MT effects were neglected in the derived mathematical model. The time evolutions of the expected value and standard deviation of the number of adsorbed particles were numerically calculated for the regime of constant analyte concentration in the reaction chamber volume, and for the regime of analyte depletion. In the former case, the sensor signal-to-noise ratio (SNR) for one fixed moment in time was analyzed, considering the effects of the sensing area reduction. However, it is well known that MT can significantly alter the sensor response kinetics [31,32,33], so it is important to consider this effect in the analysis of stochastic response. It was also experimentally shown that a suppression of MT influence leads to a great improvement of the biosensor limit of detection [34], which implies that MT affects detection limits. Therefore, fluctuations and noise models used for the estimation of the ultimate sensing performance should also include the MT influence.

In reference [27], a stochastic model of analyte diffusion within the biosensor chamber was presented, which incorporates the probabilistic model for the specific binding of analyte particles to immobilized probes at a sensing surface, as one of the boundary conditions. The model was used for the analysis of two idealized situations: (1) when there is an infinite adsorption capacity of the sensing surface, and (2) when the number of probes is finite, but a very small fraction of analyte particles present in the system is captured by the probes (i.e., no sample depletion by the binding events; thus, the number of free analyte particles in the chamber, available for adsorption, is considered constant). The closed-form solutions for the statistical parameters of biosensor response and for noise figures of merit were derived only for the biochemical equilibrium for these two cases. By using the stochastic modeling of the analyte capturing, considering the binding kinetics and the mass transfer by diffusion, the expressions for the equilibrium statistical response parameters and settling time approximation were obtained in [16]. In references [15,24,25,28], a theory was presented with closed-form expressions, as well as an analysis of the sensor AD noise power spectral density in the steady state, when the fluctuations are caused by coupled stochastic AD process and MT.

In [18,29,30], a stochastic simulation was used for the analysis of the change in the expected value and variance of the number of adsorbed particles, and the sensor signal-to-noise ratio in time, considering the transport of analyte particles by diffusion. The emphasis was on the influence of the target substance concentration and probe density on the mentioned time dependences, while MT influence was not analyzed in particular.

None of the mentioned works provided a stochastic model of sensor time response that takes into account both the diffusion and convection of analyte particles as processes that constitute MT in microfluidic sensors. In addition, none of them analyzed the MT influence neither on the stochastic temporal response, including its time-dependent expected value and variance (i.e., AD noise), nor on the SNR that determines the ultimate detection and quantification limit.

In this paper, we aim to investigate the temporal change in the statistical parameters of the biosensor stochastic response from the beginning of the adsorption process on the sensing surface until the steady state is reached, taking into account the mass transfer of analyte particles by both convection and diffusion, which corresponds to the realistic operating conditions in microfluidic biosensors. We first present the theoretical model for the expected value and variance of the number of adsorbed particles. The model is devised by applying the approach based on the master equation for the random processes known as birth–death processes in probability theory, to which the considered random process *N* belongs, and by introducing the effective probabilities of the increase and decrease in the number of adsorbed particles. The effective probabilities combine the influences of the inherently random AD and MT processes on the change in the number of adsorbed particles. By using the obtained analytical model, we investigate the response kinetics and AD noise of a protein biosensor, through the analysis of the expected value and the variance of the number of adsorbed particles, both in the transient regime and in the steady state of the binding process, for practically relevant analyte concentrations, mass transfer coefficients, and sensing surface areas. We also analyze the sensor signal-to-noise ratio, which sets the fundamental detection and quantification limits. One of the goals of our analysis is to investigate both the qualitative and the quantitative influence of MT on the kinetics of sensor stochastic response, on sensor AD noise, and on the maximal achievable SNR value, affecting both the reliable analyte detection and determination of analyte concentration. Although such an analysis can reveal new guidelines for the optimization of sensor design and operating conditions, to the best of the authors’ knowledge, it does not exist in the published literature. Another goal is to determine the applicability boundaries of the simple stochastic model of sensor response (i.e., the one that neglects the mass transfer influence), and thus the conditions under which it becomes necessary to use the more comprehensive stochastic model (that takes into account the AD process coupled with the MT of analyte particles) in order to improve the interpretation of the measurement results and the estimation of sensor performance metrics such as noise, SNR, and analyte detection and quantification limits.

## 2. Method—Mathematical Modeling of Biosensor Stochastic Time Response

The change in the number of target analyte particles adsorbed on a sensing surface in unit time, assuming reversible adsorption, is determined by the difference between the instantaneous rates of the increase and decrease in *N*(*t*), denoted by *a*(*t*) and *d*(*t*), respectively. These rates include the combined effects of adsorption–desorption and mass transfer processes. Namely, both AD and MT (convection and diffusion) processes change the target analyte concentration in a microfluidic sensor chamber, affecting the temporal change in *N*. The use of the two-compartment model (TCM) for the approximation of the spatial- and time-dependent analyte concentration in a flow-through chamber, when modeling the sensor response kinetics influenced by mass transfer, is experimentally verified for various adsorption-based biosensors [31,32,33,35]. It covers the case of surface reaction (i.e., AD process) coupled with convection and diffusion, when a thin zone (the inner compartment), depleted of analyte particles, is formed adjacent to the sensing surface, while the remaining part of the sensor chamber (the outer compartment) approximately retains the spatially uniform and time-constant analyte concentration *C*, equal to the analyte concentration in the sample introduced in the sensor chamber [31,32,33,35], as illustrated in Figure 1. By assuming the 1:1 binding of analyte molecules to the surface binding sites, the uniformity of all binding sites, and no interaction between analyte molecules, TCM yields Equation (1), which defines the temporal change in *N* [15,31,36]:

(1) $$\frac{dN}{dt}=k_{a}C_{S}(N_{m}-N)-k_{d}N=k_{a}\frac{C+\frac{k_{d}}{k_{m}A}N}{1+\frac{k_{a}}{k_{m}A}(N_{m}-N)}(N_{m}-N)-k_{d}N=a(N)-d(N)$$

where *k_a_* and *k_d_* are the adsorption and desorption rate constants, respectively, *C_S_* is the analyte concentration in the immediate vicinity of the binding sites on the sensing surface of area *A*, *N_m_* is the number of binding sites on the surface, and *k_m_* is the mass transfer coefficient, introduced in TCM as *k_m_* = 1.467(*D*^2^*v_m_*/(*L_s_h_c_*))^1/3^ [31] in order to characterize the transport of analyte particles by both convection and diffusion between the bulk solution and the surface binding sites (*D* is the diffusion coefficient of analyte particles, *v_m_* is the mean convection velocity, *L_s_* is the adsorption zone length, and *h_c_* is the sensor chamber height). According to TCM, all quantities are averaged across the sensing surface. The effective rates of the increase and decrease in the number of adsorbed particles, *a*(*t*) and *d*(*t*), respectively, do not depend explicitly on *t*, but on the instantaneous value of *N*, thus *a*(*N*) and *d*(*N*) in Equation (1).

Equation (1) is derived for the diffusion-limited regime. The equation for the case of the adsorption-limited regime (i.e., the “rapid mixing” regime, when the analyte concentration in the whole flow-through reaction chamber is considered uniform in space and constant in time, due to fast MT compared to adsorption) neglects MT effects, and it is given as:

(2) $$\frac{dN}{dt}=k_{a}C(N_{m}-N)-k_{d}N=a_{RM}(N)-d_{RM}(N),$$

where *a_RM_*(*N*) and *d_RM_*(*N*) are actual adsorption and desorption rates.

The time evolution of the number of adsorbed particles *N*(*t*) is obtained from the deterministic kinetic Equations (1) or (2) (with or without taking MT into account, respectively) for given initial conditions. It enables the analysis of the deterministic time response of a sensor, as a function of the number of adsorbed particles, which is assumed to be a deterministic quantity.

The number of adsorbed particles at any given time is a result of a sequence of random bindings and unbindings of target particles to and from the surface adsorption sites. Therefore, the number of adsorbed particles on the sensor’s active surface, *N*, is actually a stochastic quantity, determined by the stochastic nature of the AD process coupled with transport processes of analyte particles. Hence, *N* randomly fluctuates around its expected value <*N*>, and consequently, the sensor response is also a fluctuating quantity.

Observed in time, the stochastic number of adsorbed analyte particles at the sensor’s surface, *N*(*t*), is a random birth–death process [37]. By assuming that in a time interval d*t*→0, *N* can be either changed by one, or unchanged (i.e., there can be an adsorption of one particle, a desorption of one particle, or a lack of AD events), the probability distribution of the random variable *N* in an arbitrary moment of time *t* (*t* ≥ 0), *P_N_*(*n*, *t*), for the given initial state *N*(0) = *n*_0_ (here, *n*_0_ = 0 as *t* = 0 is assumed as the moment when the AD process starts on the sensing surface), is given by the master equation:

(3) $$\frac{d}{dt}P_{N}(n,t)=P_{N}(n-1,t)\cdot A(n-1)+P_{N}(n+1,t)\cdot D(n+1)-P_{N}(n,t)\cdot (A(n)+D(n))$$

where *n* is the actual value of the random variable *N* at the given moment, and it denotes the state of the process (*n*∈{0,1,2...*N_m_*}, where *N_m_* is the total number of adsorption sites on the sensing surface), while *A*(*n*)d*t* and *D*(*n*)d*t* are the probabilities of transition from the state *n* to the state *n* + 1, and from the state *n* to the state *n* − 1 during the time interval d*t*→0, respectively. *A*(*n*) and *D*(*n*) are the probability of the increase in the number of adsorbed particles by 1 in unit time, and the probability of the decrease in *N* by 1 in unit time, respectively, when the current state is *N* = *n*. Equation (3) is valid for *n* = 0 if we define *P_N_*(−1,*t*) = 0 and *A*(−1) = 0, and it is also valid for *n* = *N_m_* assuming *P_N_*(*N_m_* + 1,*t*) = 0 and *D*(*N_m_* + 1) = 0 (*D*(0) = 0 due to the nature of the desorption process, and *A*(*N_m_*) = 0 because the sensing surface adsorption capacity is limited to *N_m_*).

As mentioned in the introduction, we are interested in the expected value of *N*, which reveals the response kinetics, and in the variance of *N*, as it is a measure of the AD noise. Starting from the definitions for the first and the second moment of the random variable:

(4) $$\langle N\rangle =\sum_{n=0}^{N_{m}}nP_{N}(n,t),\;\sigma^{2}=\langle {\left(\Delta N\right)}^{2}\rangle =\sum_{n=0}^{N_{m}}{\left(n-\langle N\rangle \right)}^{2}P_{N}(n,t)$$

and using the master equation (Equation (3)), the exact equations for the expected value, <*N*>, and the variance, *σ*^2^, of the random number of adsorbed particles are obtained [37]:

(5) $$\frac{d}{dt}\langle N\rangle =\langle A(N)-D(N)\rangle$$

(6) $$\frac{d\sigma^{2}}{dt}=\langle A(N)+D(N)\rangle +2\langle \left(N-\langle N\rangle )[A(N)-D(N)\right]\rangle$$

The transition rate *A*(*N*) depends on the adsorption rate constant, on the fraction of adsorption sites available for adsorption, and on the amount of particles that are available to participate in adsorption, when the number of adsorbed particles is *N* = *n*, while *D*(*N*) depends on the desorption rate constant and on the fraction of occupied sites on the surface *N* = *n*. Thus, when the combined effect of AD and MT processes determines the probabilities of the change in the random variable *N*, the use of TCM for the approximation of the amount of particles that are available to participate in adsorption (those located in the immediate vicinity of the surface binding sites, as explained for Equation (1)) yields *A*(*n*) = *k_a_C_s_*(*N_m_* − *n*) = *k_a_*(*C* + *k_d_n*/(*k_m_A*))(*N_m_* − *n*)/(1 + *k_a_*(*N_m_* − *n*)/(*k_m_A*)) and *D*(*n*) = *k_d_n*. In this way, the expressions are obtained for the effective probabilities of the increase and decrease in the number of adsorbed particles per unit time, which combine the influences of the AD and MT processes. *A*(*n*) and *D*(*n*) depend on the current state *N* = *n* (which is a feature of birth–death processes). After representing the nonlinear transition rate as a Taylor series centered at the expected value, Equations (5) and (6) take the approximate form, which includes the first and the second moments:

(7) $$\frac{d\langle N\rangle }{dt}=A(\langle N\rangle )-D(\langle N\rangle )+\left(A^{\prime \prime }-D^{\prime \prime }\right)\cdot \frac{\sigma^{2}}{2},$$

(8) $$\frac{d\sigma^{2}}{dt}=A(\langle N\rangle )+D(\langle N\rangle )+\left[2\left(A^{\prime }-D^{\prime }\right)+\frac{1}{2}\left(A^{\prime \prime }+D^{\prime \prime }\right)\right]\cdot \sigma^{2},$$

(*A*′, *D*′, *A*″, and *D*″ are the first and second derivatives of *A* and *D* with respect to *n*, calculated for *n* = <*N*>) [38]. After substituting the functions *A* and *D* and their derivatives in Equations (7) and (8), a system of equations is obtained:

(9) $$\frac{d\langle N\rangle }{dt}=\frac{k_{a}C(N_{m}-\langle N\rangle )-k_{d}\langle N\rangle }{1+\frac{k_{a}}{k_{m}A}(N_{m}-\langle N\rangle )}-\frac{k_{a}}{k_{m}A}\frac{\frac{k_{a}k_{d}}{k_{m}A}N_{m}+k_{a}C+k_{d}}{{\left[1+\frac{k_{a}}{k_{m}A}(N_{m}-\langle N\rangle )\right]}^{3}}\cdot \sigma^{2},$$

(10) $$\frac{d\sigma^{2}}{dt}=\frac{k_{a}(C+2\frac{k_{d}}{k_{m}A}\langle N\rangle )(N_{m}-\langle N\rangle )+k_{d}\langle N\rangle }{1+\frac{k_{a}}{k_{m}A}(N_{m}-\langle N\rangle )}-\frac{\frac{k_{a}k_{d}}{k_{m}A}N_{m}+k_{a}C+k_{d}}{{\left[1+\frac{k_{a}}{k_{m}A}(N_{m}-\langle N\rangle )\right]}^{3}}\left\{2\left[1+\frac{k_{a}}{k_{m}A}(N_{m}-\langle N\rangle )\right]+\frac{k_{a}}{k_{m}A}\right\}\cdot \sigma^{2},$$

which is solved for <*N*> and *σ*^2^ (with the conditions <*N*> = 0 and *σ*^2^ = 0 at the moment *t* = 0).

The time-dependent SNR is defined as:

(11) $$SNR(t)=\frac{\langle N\rangle }{\sigma }.$$

As *σ* is a measure of fluctuations resulting from the stochastic nature of the processes (AD coupled with MT) upon which the sensor operation is based, these fluctuations constitute the fundamental, i.e., unavoidable noise. Thus, the SNR defined in this way is the best possible SNR (also known as the quantum-limited SNR in the literature [16,20]) for a given adsorption-based sensor design and parameter set.

The steady-state expected value and variance of the number of adsorbed particles according to the presented model are obtained from Equations (9) and (10), respectively, for d<*N*>/d*t* = 0 and d*σ*^2^/d*t* = 0:

(12) $$\langle N\rangle_{e}=\frac{N_{m}k_{a}C}{k_{a}C+k_{d}\left(1+\frac{k_{a}}{k_{m}A}\right)},$$

(13) $$\sigma_{e}^{2}=k_{d}\langle N\rangle_{e}\frac{{\left[1+\frac{k_{a}}{k_{m}A}(N_{m}-\langle N\rangle_{e})\right]}^{2}}{k_{a}C+k_{d}+\frac{k_{a}k_{d}N_{m}}{k_{m}A}},$$

(14) $$\sigma_{e}^{2}=k_{d}k_{a}CN_{m}\cdot \frac{{\left[k_{a}C+k_{d}\left(1+\frac{k_{a}}{k_{m}A}\right)\left(1+\frac{k_{a}}{k_{m}A}N_{m}\right)\right]}^{2}}{{\left[k_{a}C+k_{d}\left(1+\frac{k_{a}}{k_{m}A}\right)\right]}^{3}\left[k_{a}C+k_{d}\left(1+\frac{k_{a}}{k_{m}A}N_{m}\right)\right]},$$

and the steady-state SNR is:

(15) $$SNR_{e}=\frac{\langle N\rangle_{e}}{\sigma_{e}}.$$

For the rapid mixing regime (i.e., adsorption-limited binding), the mass transfer influence is neglected, and the transition probabilities per unit time are *A_RM_*(*n*) = *k_a_C*(*N_m_* − *n*) and *D_RM_*(*n*) = *k_d_n*. In that case, Equations (5) and (6) yield the system of exact equations:

(16) $$\frac{d\langle N\rangle }{dt}=A_{RM}(\langle N\rangle )-D_{RM}(\langle N\rangle ),$$

(17) $$\frac{d\sigma^{2}}{dt}=A_{RM}(\langle N\rangle )+D_{RM}(\langle N\rangle )-2\left(k_{d}+k_{a}C\right)\cdot \sigma^{2},$$

well-known in the literature [39], and whose solutions for the time evolution of both the expected value and the variance of *N* are:

(18) $$\langle N\rangle_{RM}=\langle N\rangle_{RM,e}(1-e^{-t/\tau_{RM}}),$$

(19) $$\sigma_{RM}^{2}=\sigma_{RM,e}^{2}(1-e^{-t/\tau_{RM}})(1+\frac{k_{a}C}{k_{d}}e^{-t/\tau_{RM}}).$$

Here:

(20) $$\langle N\rangle_{RM,e}=\frac{k_{a}CN_{m}}{k_{d}+k_{a}C},$$

(21) $$\sigma_{RM,e}^{2}=k_{d}\tau_{RM}\langle N\rangle_{RM,e}=\frac{k_{d}k_{a}CN_{m}}{{\left(k_{d}+k_{a}C\right)}^{2}}$$

are the expected value and the variance in the steady state, respectively, and *τ_RM_* = 1/(*k_d_* + *k_a_C*). The time-dependent SNR and its steady-state value in the case of neglected MT influence are obtained from Equations (11) and (15), respectively, by using <*N*>*_RM_*, *σ_RM_*, <*N*>*_RM_*_,*e*_, and *σ_RM_*_,*e*_ instead of the corresponding parameters of the model that includes the MT effect.

Equations (9)–(15) constitute a theoretical model that enables the investigation of the microfluidic sensor stochastic response, and also of the sensor AD noise and SNR, in the case of adsorption–desorption coupled with analyte convection and diffusion. The same quantities, but in the case of neglected MT influence, can be investigated by using the theoretical model given by Equations (11), (15), (18)–(21).

## 3. Results and Discussion

The theoretical models presented in Section 2 are used here for the investigation of statistical parameters of the stochastic response and noise performance of a biosensor for the detection of proteins in a liquid sample (the model is applicable to various receptor–ligand pairs, i.e., various biological analytes (not only proteins), whose binding to the adsorption sites can be described by Equation (1); the parameter values used in our analysis are very close to those in [31], which are within the ranges found in BIACORE experiments with proteins). As the time response of adsorption-based sensors is a function (preferably linear) of the number of adsorbed target particles, we perform the stochastic analysis of the random process *N*(*t*). That enabled us to obtain some general conclusions that are valid for the various types of adsorption sensors, regardless of their measurement parameter (optical, electrical, or mechanical, such as the refractive index, conductance, or mechanical resonance frequency), whose adsorption-induced time change constitutes the sensor response.

We first analyze and discuss the temporal change in both the expected value and the variance of the number of adsorbed particles, and of the sensor maximal achievable SNR (Section 3.1). Subsequently, we present the analysis of the same quantities after the established steady state of all the influencing transient processes (Section 3.2). Various practically relevant analyte concentrations, mass transfer coefficients, and sensing surface areas are considered.

### 3.1. Analysis of Time Evolution of the Expected Value and Variance of the Number of Adsorbed Particles and Sensor Signal-to-Noise Ratio, Considering MT Influence

**Figure 2:**
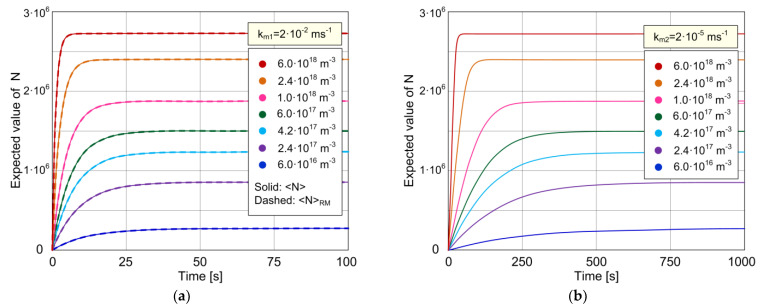
The expected value of the number of adsorbed particles in time, reflecting the kinetics of a biosensor stochastic response for different concentrations of the target protein in the analyzed sample: (**a**) The case of negligible MT influence—the curves <*N*> (shown by solid lines) according to the model that considers MT for *k_m_*_1_ = 2·10^−2^ ms^−1^ show the overlapping with the curves predicted by the model that neglects MT, <*N*>*_RM_* (dashed lines, entirely covered by solid lines). (**b**) The case of MT influenced kinetics (*k_m_*_2_ = 2·10^−5^ ms^−1^).

Figure 2a,b show the time-dependent expected value of the number of adsorbed particles, <*N*>, for different concentrations of the target protein in the sample (ranging from 6·10^16^ to 6·10^18^ m^−3^). The curves shown as solid lines in the presented diagrams are obtained by using the stochastic model given by Equations (9) and (10), which is numerically solved. The diagrams enable the investigation of the kinetics of the stochastic sensor response, considering MT effects. The AD process parameters are *k_a_* = 1.33·10^−19^ m^3^s^−1^ and *k_d_* = 0.08 s^−1^, there are *N_m_* = 3·10^6^ adsorption sites on the sensing surface of area *A* = 10^−9^ m^2^, and the mass transfer coefficients are *k_m_*_1_ = 2·10^−2^ ms^−1^ for Figure 2a and *k_m_*_2_ = 2·10^−5^ ms^−1^ for Figure 2b. All the parameter values are very close to those used in [31], for which the TCM applicability has been demonstrated in the same work.

As it can be seen in Figure 2a,b, the expected response is a monotonically increasing function of time for all concentrations, for both values of *k_m_*. The slower mass transfer process (low *k_m_*) prolongs the transient period of the time response at all concentrations, while the influence on the equilibrium expected value is not noticeable for the given set of parameter values. These conclusions are in accordance with those corresponding to the response kinetics described by the deterministic model (Equation (1)) [28,31].

The curves corresponding to the values *k_m_* > *k_m_*_1_ match those shown in Figure 2a, which means that for *k_m_* = *k_m_*_1_, mass transfer is already sufficiently fast to be of a negligible influence on the response kinetics. This explains the matching of curves (solid lines in Figure 2a), obtained by using the stochastic model that takes into account the coupling of stochastic AD and MT processes characterized by the coefficient *k_m_*_1_, with those obtained by using the stochastic model that neglects the influence of MT (dashed lines in Figure 2a, entirely covered by solid lines). Indeed, the expressions given in Section 2 show that, for a sufficiently high *k_m_*, transition rates according to the model that takes into account MT, *A*(*n*) and *D*(*n*), become equal to *k_a_C*(*N_m_* − *n*) and *k_d_n*, respectively, which are the well-known expressions for transition rates *A_RM_*(*n*) and *D_RM_*(*n*), respectively, valid when MT effects are negligible. This means that, for a sufficiently high *k_m_*, the derived stochastic model, given by Equations (9) and (10), reduces to the model presented by Equations (18) and (19), i.e., the former model is a superset of the latter. Therefore, the model that takes into account the coupling of AD and MT processes covers the cases of both the pronounced and negligible MT effects on the stochastic response, and it is in this example applied for the research of microfluidic sensor kinetics both in the case when the MT influence is significant, i.e., in the mass transfer-limited regime (as shown in Figure 2b), and in the case when the MT influence is negligible, i.e., in the regime of rapid mixing or the adsorption-limited kinetics (as shown in Figure 2a).

**Figure 3:**
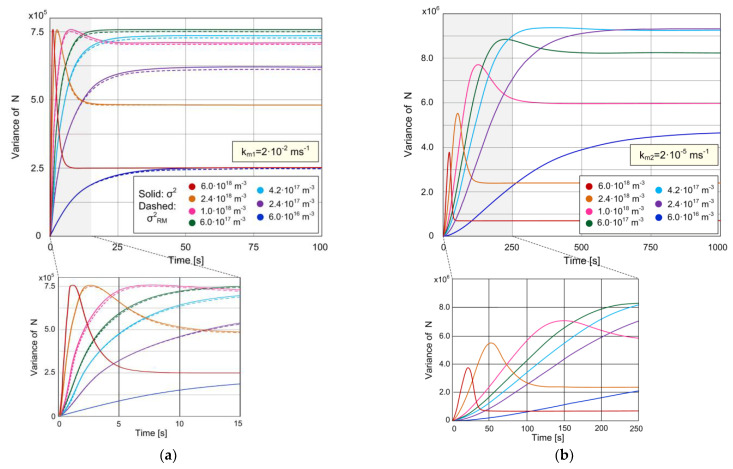
Variance of the number of adsorbed particles, revealing the behavior of the sensor response variance, i.e., sensor’s AD noise during time, for different MT coefficient values: (**a**) *k_m_*_1_ = 2·10^−2^ ms^−1^ (solid lines correspond to the stochastic model that takes into account the coupling of AD and MT processes, dashed lines correspond to the model that neglects the MT influence); (**b**) *k_m_*_2_ = 2·10^−5^ ms^−1^ (according to the model that considers the combined effects of AD and MT).

The variance, which is a measure of AD noise, is another statistical parameter of the sensor stochastic response that we analyze. Figure 3a,b show the time-dependent variance of the protein biosensor for which the expected value of the number of adsorbed particles is shown in Figure 2a,b for the same seven concentrations. The diagram in Figure 3a is obtained for *k_m_*_1_ = 2·10^−2^ ms^−1^, and it corresponds to the expected value given in Figure 2a, while the diagram in Figure 3b is for *k_m_*_2_ = 2·10^−5^ ms^−1^, and it constitutes a pair with the diagram in Figure 2b. The solid line curves in Figure 3a,b are obtained by using the theoretical model that takes into account AD and MT processes (Equations (9) and (10)), while the dashed lines in Figure 3a represent the variances determined according to the model that neglects the MT influence (Equations (18) and (19)).

The analysis of the solid line curves in Figure 3a shows that at the concentration of 6·10^17^ m^−3^ and lower, *σ*^2^(*t*) is a monotonically increasing function. As the concentration increases to 6·10^17^ m^−3^, the transient regime duration decreases, and the equilibrium variance value increases. With the further increase in the concentration, the dependence *σ*^2^(*t*) has an increasingly prominent peak, the duration of the transient regime continues to decrease, while the steady-state variance value decreases.

Figure 3b shows that slower MT causes the appearance of a peak in the dependence *σ*^2^(*t*) at lower concentrations (noticeable even at *C* = 4.2·10^17^ m^−3^). As the concentration increases, the transient regime duration decreases, and the steady-state variance value first increases and then decreases with the concentration, in the same way as in the case of high *k_m_* (Figure 3a). The peak becomes increasingly pronounced with the concentration beyond 4.2·10^17^ m^−3^, and the maximal variance (corresponding to the peak) noticeably decreases. These conclusions stemming from our model, regarding the time-dependent variance when the MT influence is pronounced, are in accordance with the results of the stochastic computer simulation, which is based on the model that takes into account coupled AD and diffusion (called “coupled hybridization-diffusion process” in the mentioned reference) in nanowire biosensors and presented in [18].

By comparing the diagrams shown in Figure 3a,b, it can be concluded that slow MT causes the increase in the variance at all the concentrations from the analyzed range. It also prolongs the time needed for the variance to reach the equilibrium value.

In Figure 3a a small difference is noticeable between the solid and dashed curves for the same concentration value. That means that *k_m_*_1_ is very close to the specific value above which the MT influence on the variance becomes negligible. For every *k_m_* value greater than that specific value, the model that takes into account MT yields the same curve *σ*^2^(*t*) as *σ_RM_*^2^(*t*) for a given *C*. When the MT influence is negligible, the analysis of Equation (19) shows that the function *σ_RM_*^2^(*t*) has a maximum (peak) at concentrations *C* > *k_d_*/*k_a_* = 6·10^17^ m^−3^, and that this maximum does not change as the concentration increases further (the peak height is independent of *C* and equal to *N_m_*/4), as can be seen in Figure 3a. At the moment when the variance is at its peak value, the expected value <*N*>*_RM_* equals *N_m_*/2 (which is obtained from Equation (18)). *σ_RM_*^2^(*t*) is a monotonically increasing function for *C* < *k_d_*/*k_a_* (then <*N*>*_RM_* <*N_m_*/2), and *σ_RM_*^2^(*t*) ≈ <*N*>*_RM_*(*t*) is valid for *C* << *k_d_*/*k_a_*. If the measurement of the signal fluctuation is used as an analytical tool in biosensing, the position and the value of the variance maximum can provide information in addition to those obtained by the noise analysis in the steady state. For example, in the case of negligible mass transfer influence, the value of the variance maximum can be used for the estimation of the number of surface binding sites *N_m_*, which is a parameter important for the estimation and optimization of the sensor performance. In addition, the existence of the variance overshoot indicates that *C* > *k_d_*/*k_a_*.

**Figure 4:**
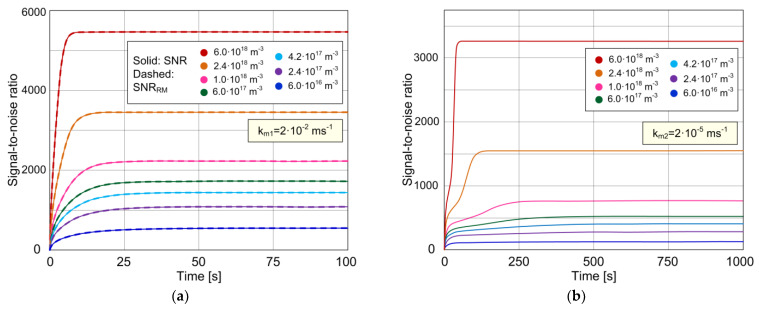
Time dependence of the sensor signal-to-noise ratio according to the model that considers the combined AD and MT effects (solid lines), for two different values of the MT coefficient: (**a**) *k_m_*_1_ = 2·10^−2^ ms^−1^; (**b**) *k_m_*_2_ = 2·10^−5^ ms^−1^. The curves obtained according to the model that neglects MT (dashed lines) match those predicted by the model that takes into account the coupling of AD and MT processes for *k_m_*_1_ (solid lines), as shown in (**a**).

Figure 4a,b show the time dependence of the best possible SNR (as defined in Equation (11)) of the biosensor in the case of the nearly negligible mass transfer influence (*k_m_*_1_), and in the case when the mass transfer influence is pronounced (*k_m_*_2_), respectively, for different analyte concentrations (the values of all parameters are given at the beginning of Section 3.1, and they are the same as for Figure 2a,b and Figure 3a,b). The curves corresponding to the cases when the MT influence is negligible (obtained according to the model that neglects MT, given by Equations (18) and (19)) are so close to those shown in Figure 4a (for *k_m_*_1_ = 2·10^−2^ ms^−1^) that the difference between them is not noticeable in the diagram of that scale. The diagrams show that the SNR decreases with the decrease in *C*, for every *t*, both for rapid and slow mass transfer. Mass transfer increases the time needed for SNR to achieve its maximum value (corresponding to the steady state) at a given concentration. In addition, slow MT decreases the SNR for the given analyte concentration. Therefore, it depends on the value of *k_m_* whether or not it is possible to reach the required SNR for reliable detection and quantification of an analyte by using a given sensor with a given set of parameter values.

### 3.2. Analysis of MT Influence on the Sensor Stochastic Response and Noise Performance in Steady State

Here, we show the analysis of the expected value and variance of the number of adsorbed particles after the steady state of all relevant transient processes has been reached. We also present the steady-state analysis of the maximal achievable SNR. In the following diagrams, denoted with solid lines are the curves obtained by using the model (given by Equations (12) and (13)) that considers fluctuations in the number of adsorbed particles as a consequence of the coupling of stochastic AD process and the mass transfer of analyte particles. The curves corresponding to the stochastic model that neglects MT (Equations (20) and (21)) are denoted with dashed lines. When MT is sufficiently fast, its effects become negligible, as shown by the matching of the corresponding expressions for the steady-state statistical parameters determined by using the two mentioned stochastic models, for the high enough values of *k_m_*. The difference between the corresponding quantities determined by using the two models can thus be used as a measure of MT influence. The MT with the coefficient *k_m_* = 2·10^−5^ ms^−1^ is assumed, the adsorption sites surface density is *n_m_* = *N_m_*/*A* = 3·10^15^ m^−2^ for all analyzed sensors, and the remaining parameter values are those given at the beginning of Section 3.1, unless otherwise noted.

**Figure 5:**
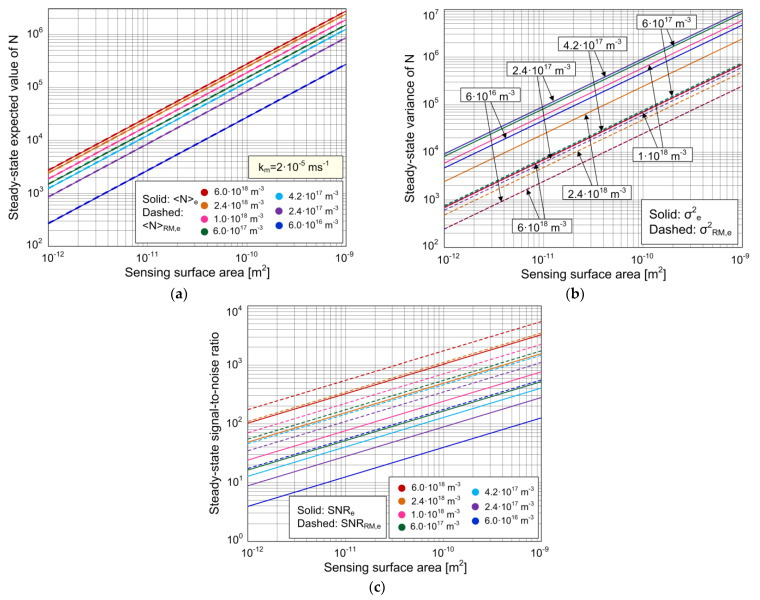
Expected value (**a**) and variance (**b**) of the number of adsorbed particles in the steady state, and the steady-state signal-to-noise ratio (**c**), as a function of the sensing surface area, for different concentrations of the target protein, according to the stochastic model that takes into account the combined effect of AD and mass transfer processes (solid lines), and according to the stochastic model that neglects mass transfer (dashed lines).

Figure 5a shows the expected value of the number of adsorbed particles in the steady state, as a function of the sensing surface area ranging from 10^−12^ to 10^−9^ m^2^, for different concentrations of the target protein. The curves obtained according to the two stochastic models match, which means that the influence of MT on the steady-state expected value is negligible for the given set of parameter values. The expressions for the steady-state expected value according to the two models, given by Equations (12) and (20), yield the ratio:

(22) $$\frac{{\langle N\rangle }_{RM,e}}{{\langle N\rangle }_{e}}=1+\frac{k_{a}/(k_{m}A)}{1+k_{a}C/k_{d}}$$

and, thus, the condition at which MT does not influence the expected number of adsorbed particles (when <*N*>*_RM_*_,*e*_/<*N*>*_e_* ≈ 1 is valid):

(23) $$k_{m}\gg \frac{k_{a}/A}{1+k_{a}C/k_{d}}=k_{m,ev}.$$

This is also the condition for the applicability of the simpler stochastic model that does not take MT into account. The most stringent condition for a given *k_m_* corresponds to the case when *k_m_*_,*ev*_ has the highest value, i.e., at the lowest analyte concentration, and in sensors of the smallest sensing surface area from the considered range. For the sets of parameter values used in our analysis (Figure 5a), the maximal *k_m_*_,*ev*_ equals 1.2·10^−7^ ms^−1^, so the most stringent condition for the expected values obtained according to the two models to be approximately equal is *k_m_* >> 1.2·10^−7^ ms^−1^, which is fulfilled for *k_m_* = 2·10^−5^ ms^−1^. This explains the matching of the curves obtained by using the two models (Figure 5a). The condition (23) is satisfied in a wide range of parameter values. However, in sensors with extremely small sensing surfaces (such as nanowire or carbon nanotube mechanical or electrical sensors), as well as in detection of particles present in ultra-low concentrations, the value of *k_m_*_,*ev*_ can be such that the condition given by Equation (23) is not satisfied, which implies that MT could influence the expected value of the sensor stochastic response by decreasing it. This conclusion is in accordance with the result of a computer simulation performed for a nanowire biosensor in [29], which showed the decrease in the expected value of the number of adsorbed particles in the case of binding influenced by diffusion. This deserves further investigation by using a model particularly considering nanoscale sensors.

The dependence of the steady-state variance of the number of adsorbed particles on the sensing surface area, determined according to the two stochastic models, is shown in Figure 5b. The target protein concentration is used as the parameter for the shown curves.

Although the curves in Figure 5a for the same sets of parameter values show a negligible difference between the steady-state expected values obtained by using the two models, the corresponding steady-state variances differ significantly, which can be seen in Figure 5b. The diagram shows that MT with the coefficient *k_m_* = 2·10^−5^ ms^−1^ causes a significant increase in the steady-state variance at a given concentration and sensing surface area. The condition for MT to be of negligible influence on the variance is obtained from *σ*^2^*_e_* ≈ *σ*^2^*_RM,e_*. It can be formulated through the ratio:

(24) $$\frac{\sigma_{e}^{2}}{\sigma_{RM,e}^{2}}=\frac{{\left(k_{a}C+k_{d}\right)}^{2}{\left[k_{a}C+k_{d}\left(1+\frac{k_{a}}{k_{m}A}\right)\left(1+\frac{k_{a}n_{m}}{k_{m}}\right)\right]}^{2}}{{\left[k_{a}C+k_{d}\left(1+\frac{k_{a}}{k_{m}A}\right)\right]}^{3}\left[k_{a}C+k_{d}\left(1+\frac{k_{a}n_{m}}{k_{m}}\right)\right]}\approx 1,$$

which is obtained from Equations (12), (13), (20) and (21). The condition (24) is more complex than the condition for approximately equal expected values (Equation (23)). By analyzing the ratio of the steady-state variances, the following ultimate condition for *k_m_* is obtained, under which the ratio approximately equals 1:

(25) $$\frac{\frac{k_{a}}{k_{m}A}+\frac{k_{a}n_{m}}{k_{m}}+\frac{k_{a}^{2}n_{m}}{k_{m}^{2}A}}{1+\frac{k_{a}C}{k_{d}}}\langle \langle 1.$$

When the condition given by Equation (25) is not satisfied, it is necessary to use the stochastic model that takes into account MT in order to perform the steady-state variance (i.e., AD noise) analysis. None of the combinations of *C* and *A* values from the considered range can satisfy the condition (25).

Figure 5b shows that the variance increases with a greater sensing surface area in cases when the MT influence is significant (solid lines), as well as in cases when MT is sufficiently fast and is thus of negligible influence (dashed lines). It can be seen from the diagram that solid and dashed lines that correspond to the same *C* value are parallel, which means that the ratio of variances determined according to the two models at the same concentration is independent of *A* in the whole considered range of *A*. In other words, the amount of MT influence on the change in steady-state variance (expressed as *σ*^2^*_e_*/*σ*^2^*_RM,e_*) does not depend on *A*. Indeed, the analysis of the expression *σ*^2^*_e_*/*σ*^2^*_RM,e_* shows that for *A* >> *k_a_*/*k_m_* = 6.65·10^−15^ m^2^ (satisfied for all *A* values within the considered range), the following is valid:

(26) $$\frac{\sigma_{e}^{2}}{\sigma_{RM,e}^{2}}\approx 1+\frac{k_{a}n_{m}/k_{m}}{1+k_{a}C/k_{d}},$$

yielding the condition for the value of *k_m_* at which the MT influence on the variance is insignificant, i.e., the applicability condition for the simpler stochastic model:

(27) $$k_{m}\rangle \rangle \frac{k_{a}n_{m}}{1+k_{a}C/k_{d}}=k_{m,var}.$$

The condition given by Equation (27) can be easiest to satisfy at the greatest *C* value from the considered range, i.e., *C* = 6·10^18^ m^−3^, but even then, *k_m_*_,*var*_ ≈ 3.6·10^−5^ ms^−1^. Due to that, the variances determined according to the two models differ significantly when the MT coefficient equals 2·10^−5^ ms^−1^, for every *C* value from the considered range.

Figure 5c shows the biosensor steady-state SNR obtained by using the two stochastic models, as a function of the sensing surface area, where the target analyte concentration is a parameter. The SNR decreases as the sensing surface area decreases (the standard deviation also decreases, but the decrease in the expected value is more pronounced, as it can be seen in Figure 5a,b), and also when the target protein concentration is lower, according to both models. A significant difference between the SNRs determined by the two models can be seen in the diagram. The decrease in the steady-state SNR, caused by MT, exists at all considered values of *C* and *A*. The condition under which the MT influence on the SNR is negligible is given by Equation (27) for the cases analyzed here (as *A* >> *k_a_*/*k_m_* = 6.65·10^−15^ m^2^ is valid). By using the model that takes into account the MT influence, and for *k_m_* that satisfies the condition (27), the curves are obtained that match those shown as dashed lines in Figure 5c.

The value of *k_m_* influences the maximal achievable SNR value of a sensor with a given sensing surface area. The diagram shown in Figure 5c can be used to determine whether or not it is possible to achieve an SNR value required to reliably detect and quantify the target substance concentration using a sensor of a given surface area. It can be seen that the steady-state SNR of a sensor with the sensing surface area of 10^−12^ m^2^, protein concentration of 6·10^16^ m^−3^, and MT coefficient of 2·10^−5^ ms^−1^ approximately equals 3, which is the minimal value needed for protein detection [40,41]. The same sensor in the case of negligible MT influence has an SNR of almost 20, so it satisfies the more stringent condition (SNR ≥ 10 [40]) for reliable quantification of the concentration. A sensor with a sensing surface area of 10^−11^ m^2^ in the case of *k_m_* = 2·10^−5^ ms^−1^ has an SNR higher than 10, so it satisfies the conditions for both analyte detection and quantification of the concentration even when the MT influence is pronounced. The same diagram enables the determination of the minimal detectable and measurable concentrations (i.e., the fundamental detection and quantification limits, as they are determined by the fundamental noise) of the given sensor, for the required SNR value (e.g., in [42], SNR = 1 was used for the estimation of the minimum detectable change in the measured quantity in a graphene ISFET, as the theoretical limit of performance determined by intrinsic noise; in [43], SNR = 1 was also used for the determination of the detection threshold in silicon nanowire sensors). These results show that the fundamental detection and quantification limits depend on the MT rate.

**Figure 6:**
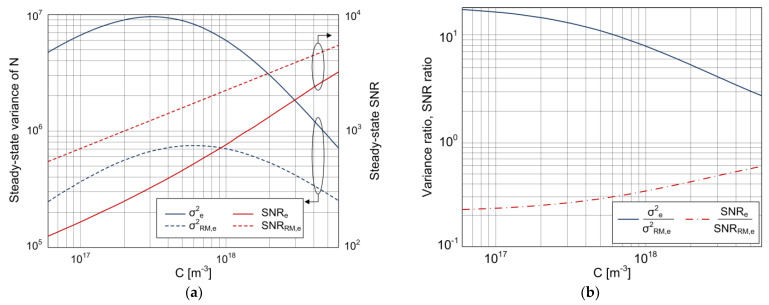
(**a**) Dependence of the variance of the number of adsorbed particles and sensor’s SNR (*A* = 10^−9^ m^2^) in the steady state on the target substance concentration, according to the stochastic model that takes into account MT (solid lines), and the model that neglects it (dashed lines). (**b**) Ratios of steady-state variances (*σ*^2^*_e_*/*σ*^2^*_RM,e_*) and SNRs (*SNR_e_*/*SNR_RM,e_*) according to the two models, depending on the concentration.

A diagram that enables the steady-state analysis of the dependences of both the sensor’s time response variance and SNR on the target substance concentration, in the cases of pronounced and negligible MT influence, is given in Figure 6a. Figure 6b shows the ratio of variances when the MT influence exists, and when it is negligible, and the ratio of SNRs for the same two cases. The curves in Figure 6a,b are for a sensor with *A* = 10^−9^ m^2^, and they correspond to the steady-state values of time-dependent variances and SNRs shown in Figure 3 and Figure 4, respectively. For the remaining surface areas considered in Section 3.2 (from 10^−12^ to 10^−10^ m^2^), the conclusions will be the same as those obtained based on Figure 6b about the MT-influenced change in the variance and SNR (expressed through the ratios *σ*^2^*_e_*/*σ*^2^*_RM,e_* and *SNR_e_*/*SNR_RM,e_*) in the considered concentration range. This is because the analysis given in the comment for Figure 5b,c showed that the magnitude of the MT influence on these two quantities does not depend on the active surface area when *A* >> *k_a_*/*k_m_* = 6.65·10^−15^ m^2^ (then, the ratios *σ*^2^*_e_*/*σ*^2^*_RM,e_* (Equation (26)) and *SNR_e_*/*SNR_RM,e_* do not depend on *A*). In addition, as *σ*^2^*_RM,e_* is proportional to *A*, and it can be shown that for surface areas *A* >> *k_a_*/*k_m_* = 6.65·10^−15^ m^2^, *σ*^2^*_e_* is also proportional to *A*, all conclusions about the dependences of *σ*^2^*_e_* and *σ*^2^*_RM,e_* on *C*, based on Figure 6a, will be valid for all sensing surface areas in the range from 10^−12^ to 10^−9^ m^2^. For a similar reason (*SNR_e_* and *SNR_RM,e_* are proportional to *A*^1/2^), conclusions based on Figure 6a about the influence of both MT and target substance concentration on the change in SNR of a sensor with the sensing surface area of 10^−9^ m^2^ are valid for other sensors of different sensing surface areas from the mentioned range.

Figure 6a shows that the steady-state variance obtained by using the model that takes into account the AD process, diffusion, and convection has a maximum at a certain concentration. This result for the MT-influenced binding of target particles is in accordance with the computer simulation results obtained for the variance of silicon nanowire field-effect biosensors whose response fluctuates due to the coupling of the AD process and mass transfer by diffusion [29,30].

In Figure 6a, it can be seen that MT influences the concentration value at which AD noise (i.e., variance) has its maximum. When MT is negligible (dashed line in Figure 6a), the variance reaches its maximum at *C_max_*_,*RM*_ = *k_d_*/*k_a_* = 6·10^17^ m^−3^. Starting from the expression for *σ*^2^*_e_* (Equation (13)), which is simplified under the condition *A* >> *k_a_*/*k_m_*, it can be analytically shown that the variance influenced by MT has the maximum at the concentration *C_max_* ≥ *C_max,RM_*. Thus, due to the influence of MT, the AD noise maximum moves toward lower concentrations of the target substance.

Figure 6b shows that, for a given sensor, the influence of MT on the increase in variance becomes more pronounced at lower analyte concentrations. As the concentration decreases, the ratio *σ*^2^*_e_*/*σ*^2^*_RM,e_*, given by Equation (26), asymptotically approaches the maximum value 1 + *k_a_n_m_*/*k_m_*≈21, while, when *C* increases, the ratio of variances approaches 1 (i.e., for given *k_m_*, the variances according to the two models are approximately equal at a sufficiently high concentration).

The dependences *SNR_e_*(*C*) and *SNR_RM,e_*(*C*), shown in Figure 6a, increase monotonically as the concentration increases. The influence of MT on the decrease in the sensor’s steady-state SNR is concentration-dependent. This can be quantitatively analyzed based on the diagram shown in Figure 6b. MT causes the greatest decrease in the sensor’s SNR at low concentrations. With the decrease in concentration, the ratio *SNR_e_*/*SNR_RM,e_* asymptotically approaches its minimum.

The dependence *SNR_e_*(*C*) obtained by using the derived analytical expression and shown in Figure 6a is in accordance with that obtained in [30] by computer simulation for the case of diffusion-influenced binding. Diagrams of this kind (Figure 6a) enable the determination of the concentration detection and quantification limits for a given sensor and given experimental conditions, as the values of *C* at which the SNR has the minimal required values for reliable analyte detection and quantification, respectively.

## 4. Conclusions

A theoretical model was presented that enables efficient analysis of the stochastic time response and ultimate noise performance of adsorption-based microfluidic chemical and biological sensors, taking into account the influence of mass transfer (MT) of the analyte particles. It was shown that for sufficiently fast MT, the model we devised match the commonly used model that neglects mass transfer, so it is applicable in a wider parameter range, covering the cases of both pronounced and negligible MT influence.

Two models (one that neglects, and the other that takes into account MT effects) were used for the analysis of statistical parameters of the protein biosensor stochastic response, for various analyte concentrations, mass transfer coefficients, and sensing surface areas. The sensor signal-to-noise ratio (SNR), which sets the fundamental detection and quantification limit, was also investigated. The comparison of results obtained according to the two stochastic models has led to the conclusions about the qualitative and quantitative influence of MT on the sensor response kinetics and noise performance metrics, both in the transient regime and in steady state.

The analysis showed that MT can significantly alter the time dependence of the expected value and variance of the number of adsorbed particles, and thus the response kinetics, adsorption–desorption (AD) noise, and SNR of the sensor.

Slow mass transfer decelerates the response kinetics. The analysis indicated that MT can also influence the steady-state response value by decreasing it, in sensors of extremely small sensing surface areas, or when particles present in ultra-low concentrations need to be detected. This secondary effect of MT, which degrades the sensor’s sensitivity and may cause false negative/positive results when interpreting measurement data, should be further investigated by using a model considering nanoscale sensors.

According to the analysis results, MT prolongs the time needed for the variance to achieve the steady-state value. Both stochastic models predict a peak in the time dependence of the variance, at concentrations greater than a certain critical value. However, MT influences the appearance of the peak in the variance transient regime at lower analyte concentrations. In the case of pronounced MT, the height of the peak decreases with the concentration. When MT is negligible, the peak height remains constant for different concentrations. The condition was formulated under which MT influences the variance in the steady state. MT can significantly increase the steady-state variance, i.e., the sensor AD noise. This effect is not limited to extremely small sensing surface areas, and it is especially pronounced at low analyte concentrations. MT also shifts the maximum of the steady-state AD noise toward the lower analyte concentrations.

An important conclusion of the analysis is that MT influences the sensor’s maximal achievable SNR value. Slow MT decreases the SNR, and therefore, for the given sensor and target substance, it depends on MT parameters whether or not it it possible to reach the required SNR value for reliable detection and quantification of an analyte in the concentration range of interest. Another effect of MT is that the maximum SNR value (i.e., the steady-state value) is reached more slowly at slower MT. The MT influence on the SNR is more pronounced at lower analyte concentrations. The results have shown that even when mass transfer does not influence the expected value, it can significantly influence both the variance of the response (which is a measure of the inevitable adsorption–desorption noise) and the signal-to-noise ratio (which sets the fundamental detection and quantification limits of adsorption-based sensors). Therefore, mass transfer can exhibit a significant influence on the ultimate sensor performance, including the minimal detectable and quantifiable analyte concentrations.

The steady-state analysis of the difference between the corresponding quantities determined by using the two stochastic models also enables the obtaining of the applicability conditions for the simpler stochastic model that does not take into account MT. At the same time, these conditions reveal the criteria that can be used to establish when it becomes necessary to apply the model that takes into account the coupling between the AD process and mass transfer. It is shown that when the sensor’s noise performance analysis is intended, the application of the stochastic model that takes MT into account may be necessary for the set of parameter values at which the simpler stochastic model is applicable for the analysis of the response kinetics.

To the best of the authors’ knowledge, these are the first results regarding the influence of MT on the sensor stochastic response and its noise characteristics. The results have illustrated the significance of the presented theoretical model for the correct interpretation of measurement results, for the estimation of sensors’ noise performance metrics important for reliable analyte detection and quantification, as well as for sensor optimization in terms of the lower detection and quantification limits. The presented model is also a useful tool for the development of new methods for the detection and quantification of substances, based on the analysis of sensor signal fluctuations. In general, the use of the stochastic model that takes into account MT becomes necessary as the sensing surface area and analyte concentration decrease. Due to the ongoing efforts toward the miniaturization of sensing devices, and the lower detectable concentrations in the latest sensor generation, it is expected for this theoretical model to be increasingly useful.

## Data Availability

The data presented in this study are available on request from the corresponding author.
